# Photocatalytic degradation properties of α-Fe_2_O_3_ nanoparticles for dibutyl phthalate in aqueous solution system

**DOI:** 10.1098/rsos.172196

**Published:** 2018-04-11

**Authors:** Yue Liu, Nan Sun, Jianshe Hu, Song Li, Gaowu Qin

**Affiliations:** 1 Center for Molecular Science and Engineering, College of Science, Northeastern University, Shenyang 110819, People's Republic of China; 2 School of Materials Science and Engineering, Northeastern University, Shenyang 110819, People's Republic of China

**Keywords:** α-Fe_2_O_3_, nanoparticles, photocatalytic degradation, dibutyl phthalate

## Abstract

The phthalate ester compounds in industrial wastewater, as kinds of environmental toxic organic pollutants, may interfere with the body's endocrine system, resulting in great harm to humans. In this work, the photocatalytic degradation properties of dibutyl phthalate (DBP) were investigated using α-Fe_2_O_3_ nanoparticles and H_2_O_2_ in aqueous solution system. The optimal parameters and mechanism of degradation were discussed by changing the morphology and usage amount of catalysts, the dosage of H_2_O_2_, pH value and the initial concentration of DBP. Hollow α-Fe_2_O_3_ nanoparticles showed the highest degradation efficiency when 30 mg of catalyst and 50 µl of H_2_O_2_ were used in the DBP solution with the initial concentration of 13 mg l^−1^ at pH = 6.5. When the reaction time was 90 min, DBP was degraded 93% for the above optimal parameters. The photocatalytic degradation mechanism of DBP was studied by the gas chromatography–mass spectrometry technique. The result showed that the main degradation intermediates of DBP were *ortho*-phthalate monobutyl ester, methyl benzoic acid, benzoic acid, benzaldehyde, and heptyl aldehyde when the reaction time was 2 h. DBP and its intermediates were almost completely degraded to CO_2_ and H_2_O in 12 h in the α-Fe_2_O_3_/ H_2_O_2_/UV system.

## 1. Introduction

As is well known, dibutyl phthalate (DBP) has been widely used as an excellent plasticizer in different resins, especially poly(vinyl chloride) resins and nitrocellulose [1,2]. In addition, DBP is also an important additive in special paints and adhesives. As DBP is only physically bound to the plastic structure, it is easily released into the natural environment [3,4]. It leads to a sharp increase in the content of DBP in our living environment. However, DBP is a rather stable compound in the natural environment [5,6]. Its hydrolysis half-life is about 20 years. The people employed in the production of this plasticizer for a long time might suffer from multiple neuritis, spinal neuritis and multiple cerebral neuritis. Therefore, it is very necessary and important to study an efficient method for the degradation of DBP in wastewater.

The conventional methods, including electrochemical method [7], chemical adsorption method [8], adsorption method [9], biodegradation method [10], only transfer the organic pollutants from one phase to another, which do not degrade [11]. However, catalytic oxidation can overcome the shortcomings of these traditional methods. So the development of photocatalytic degradation technology has brought new potential for the treatment of environmental pollutants and energy crisis response. At present, the photocatalytic degradation technology has been the most active research field in waste water, waste gas purification and hydrogen production [12–15].

As an important photocatalytic material, α-Fe_2_O_3_ exhibits good properties [16], such as low-cost synthesis, no secondary pollution [17], n-type semiconducting behaviour and band gap (*E*_g_ = 2.1 eV) [18]. It has attracted considerable attention due to its potential application in the fields of sensors, pigments, actuators, catalysts and so on [19–21].

Until now, there have been numerous reports on photocatalytic degradation of environmental pollutants by TiO_2_ [22–24]. By contrast, there is little research on the photocatalytic degradation by Fe_2_O_3_ of organic pollutants. Moreover, its corresponding reaction mechanism has not been explained in detail. Compared with those of TiO_2_, the valence electrons of Fe_2_O_3_ can be excited by less than 560 nm to the conduction band; this can greatly improve the efficiency of the use of sunlight [25].

In previous work, we reported α-Fe_2_O_3_ nanoparticles with different morphologies [26]. In this study, the photocatalytic effect of α-Fe_2_O_3_ nanoparticles was evaluated through the degradation of DBP. The effect of the operating parameters, such as the morphology and dosage of Fe_2_O_3_, the initial concentration of H_2_O_2_, the initial concentration of DBP and the pH of solution, on the degradation efficiency was discussed. The photocatalytic reaction mechanism and degradation process of DBP over α-Fe_2_O_3_ were investigated with gas chromatography–mass spectrometry (GC-MS).

## 2. Experimental

### 2.1. Materials

DBP was purchased from Shenyang Li Cheng Reagent plant (Shenyang, China). Hydrogen peroxide (30%) was purchased from Shenyang Xinxing Chemical Reagent plant (Shenyang, China). The α-Fe_2_O_3_ nanoparticles with different morphologies were prepared in our laboratory [26]. All other solvents and reagents used were purified by standard methods.

### 2.2. Characterization

The absorption wavelength was measured by a TU-1901 dual-beam UV–visible spectrophotometer (Beijing Purkinje General Instrument Co. Ltd, China) with a range of 450–600 nm. The degradation products of DBP were analysed by GC-MS (HP6890s/HP5973 GC/MS, Perkin-Elmer, Norwalk, USA).

### 2.3. Photocatalytic degradation experiments

**Figure 1.**
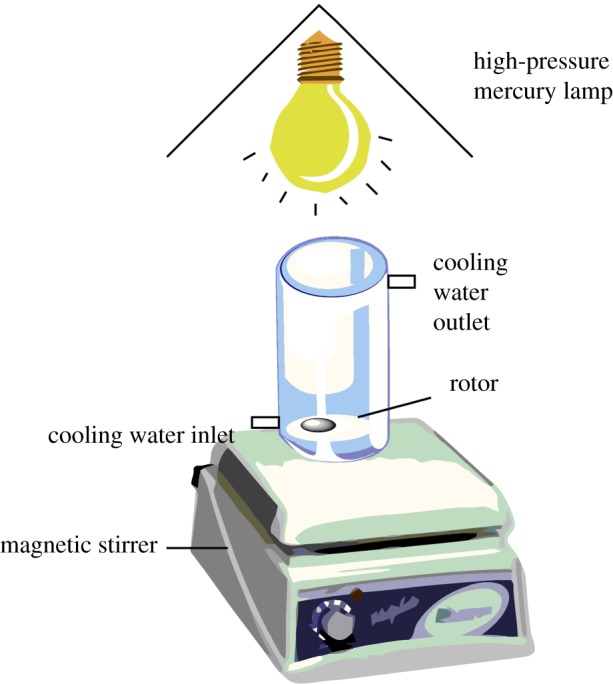
Reactor of photocatalysis for DBP.

The photocatalytic degradation experiments were carried out in a homemade photocatalytic reactor. The experimental set-up is shown in figure 1. A high-pressure mercury lamp was used as a light (250 W) source, the distance between the reactor and the light was 20 cm, and the reactor was passed through the condensate to ensure that the entire reaction took place under a constant temperature.

The DBP aqueous solution (100 ml) was placed in the photocatalytic reactor, and then the appropriate amount of Fe_2_O_3_ powder and a certain volume of 30% H_2_O_2_ were added to conduct the photocatalytic degradation process. The photocatalytic degradation process of DBP over α-Fe_2_O_3_ was carried out by changing the amount of Fe_2_O_3_, H_2_O_2_ content, pH and initial concentration of DBP.

## 3. Results and discussion

### 3.1. Establishment of the standard curve

The UV–visible spectra of the different concentrations of DBP solution are shown in electronic supplementary material, figure S1. The scanning range was 190–350 nm. The characteristic absorption wavelength of DBP was 230 nm, so that wavelength was selected. The relationship between the mass concentration of DBP solution and the absorbance is shown in electronic supplementary material, figure S2. From the standard curve, the absorbance value at the characteristic absorption wavelength showed a good linear relationship in the DBP solubility range, and the following regression equation was obtained from the standard curve:

3.1 $$A=0.023c+0.003\;R^{2}=0.99,$$

where *A* is the absorbance and *c* is the mass concentration of DBP solution. According to the standard curve, the relationship between the mass concentration and the absorbance was in accordance with the Lambert–Beer rule. Therefore, the degradation efficiency in this concentration range could be expressed by the following formula:

3.2 $$K=\frac{A_{0}-A_{t}}{A_{0}}\times 100\%,$$

where *K* is the degradation rate, *A*_0_ is the absorbance of the initial solution and *A_t_* is the absorbance of the solution at time *t*.

### 3.2. Effect of α-Fe_2_O_3_ particles with different morphology on degradation of dibutyl phthalate

**Figure 2.**
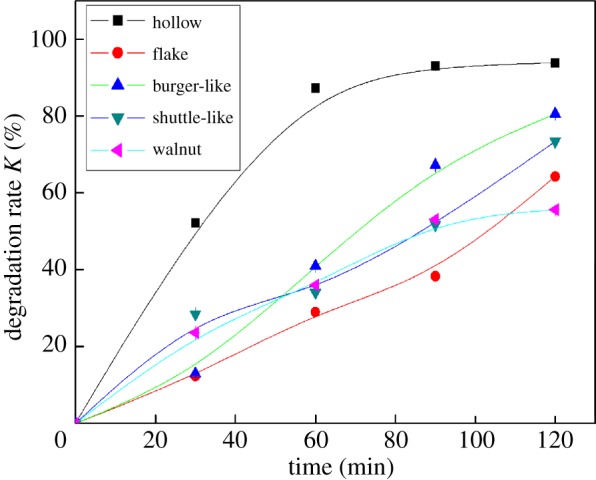
Effect of different morphologies of Fe_2_O_3_ on the degradation efficiency of DBP (Fe_2_O_3_, 30 mg; 30% H_2_O_2_, 50 µl; pH = 6.5; temperature, 25°C).

The morphology of α-Fe_2_O_3_ particles is an important factor affecting degradation processes, so it is necessary to explore the effect of α-Fe_2_O_3_ particles with different morphology on degradation of DBP. Figure 2 shows the photocatalytic activity of α-Fe_2_O_3_ with different morphologies for the degradation of DBP. It could be clearly seen that the degradation efficiency varied with α-Fe_2_O_3_ morphology. Among them, α-Fe_2_O_3_ nanoparticles with hollow morphology had the highest degradation efficiency. The possible reason was that the structure of hollowα-Fe_2_O_3_ nanoparticles could produce more hydroxyl radicals (•OH). In addition, the dispersion of hollow α-Fe_2_O_3_ nanoparticles and the specific surface area were larger than those of other forms of nanoparticles, so the most effective reaction area was obtained. Based on the results shown in figure 2, the hollow morphology was optimal and selected for further studies.

### 3.3. Effect of α-Fe_2_O_3_ dosage on the degradation of dibutyl phthalate

**Figure 3.**
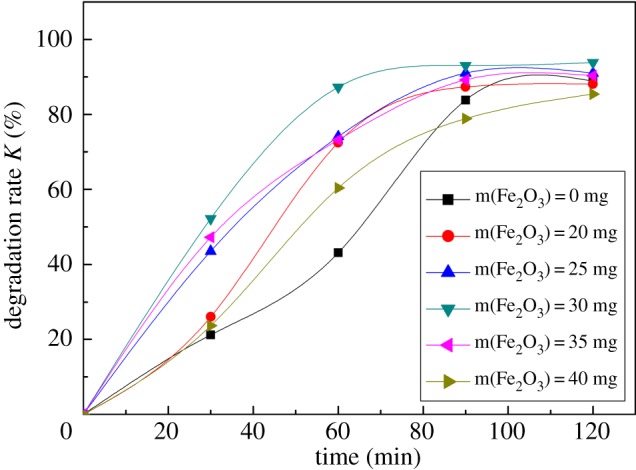
Effect of Fe_2_O_3_ content on the degradation efficiency of DBP (13 mg l^−1^ DBP, 100 ml; pH = 6.5; temperature, 25°C).

The catalyst dosage is also an important factor affecting degradation processes, so it is necessary to explore the effect of α-Fe_2_O_3_ dosage on the degradation efficiency of DBP. To determine the optimal dosage, various amounts of α-Fe_2_O_3_ nanoparticles were tested. Figure 3 shows the degradation of DBP with α-Fe_2_O_3_ dosage. As seen, the degradation efficiency of DBP solution was the highest when the amount of Fe_2_O_3_ was 30 mg/100 ml. This phenomenon was explained as follows. When the effective area of the photocatalytic reaction increased, the amount of photo-generated electron–hole pairs and hydroxyl radicals also increased. This indicated that the degradation rate of DBP was strongly influenced by the number of active sites and photon absorption ability of the α-Fe_2_O_3_ nanoparticles. However, when the amount of the α-Fe_2_O_3_ nanoparticles was too much, the propagation of the incident light in the reaction solution was hindered. At this moment, the light scattering effect increased so that the light transmittance reduced, which affected the photocatalytic degradation efficiency. Based on the results shown in figure 3, the optimal dosage was 30 mg/100 ml.

### 3.4. Effect of H_2_O_2_ content on degradation of dibutyl phthalate

As is well known, H_2_O_2_ can be photolysed to produce hydroxyl radical group under light irradiation, the reaction being described as follows:

3.3 $${\text{H}}_{2}{\text{O}}_{2}\to 2∙\text{OH}.$$

The hydroxyl radical group plays a critical role in the degradation process of organics: it can be involved in hydroxyl substitution reaction, dehydrogenation reaction or electron transfer reaction, leading to sensitization and degradation of organics.

**Figure 4.**
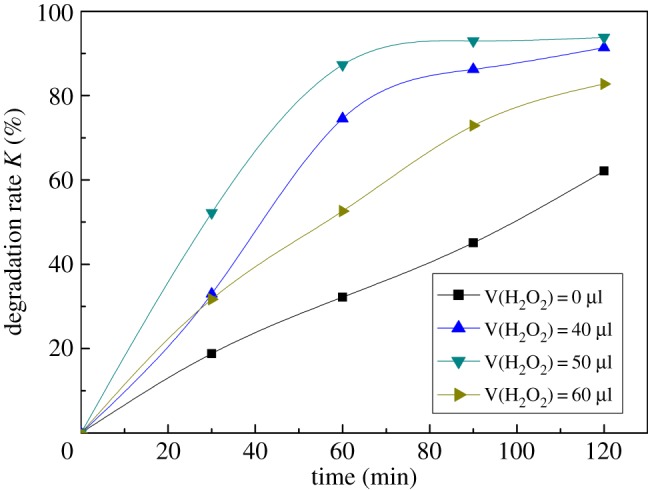
Effect of H_2_O_2_ content on the degradation efficiency of DBP (13 mg l^−1^ DBP, 100 ml; Fe_2_O_3_, 30 mg; pH = 6.5; temperature, 25°C).

Figure 4 shows the effect of H_2_O_2_ content on the degradation efficiency of DBP. As seen in figure 4, the degradation efficiency of DBP solution was the highest when H_2_O_2_ content was 50 µl. In the photocatalytic reaction system, the addition of H_2_O_2_ can increase the rate of hydroxyl radical formation. At the same time, H_2_O_2_ is also an electron capture agent that can inhibit the complex effects of photo-generated electron–hole pairs. So the addition of H_2_O_2_ could accelerate the photocatalytic degradation efficiency of DBP. When the amount of H_2_O_2_ was less, the •OH produced in the reaction system was less, so the degradation efficiency was low. With the increase of H_2_O_2_ content, the formation rate of •OH in the system also increased, so the photocatalytic degradation efficiency was obviously improved. However, when the H_2_O_2_ addition exceeded the critical value, the generation of •OH was inhibited because excessive H_2_O_2_ had a capture effect on •OH, resulting in a decrease in degradation efficiency. The reaction is described as follows:

3.4 $$\begin{array}{c} ∙\text{OH}+{\text{H}}_{2}{\text{O}}_{2}\to {\text{H}}_{2}\text{O}+\text{H}{\text{O}}_{2}. \end{array}$$

3.5 $$\begin{array}{c} ∙\text{OH}+\text{H}{\text{O}}_{2}∙\to {\text{O}}_{2}+{\text{H}}_{2}\text{O}. \end{array}$$

3.6 $$\begin{array}{c} ∙\text{OH}+∙\text{OH}\to {\text{H}}_{2}{\text{O}}_{2}. \end{array}$$

According to the results shown in figure 4, the optimal 30% H_2_O content was 50 µl.

### 3.5. Effect of solution pH on degradation of dibutyl phthalate

**Figure 5.**
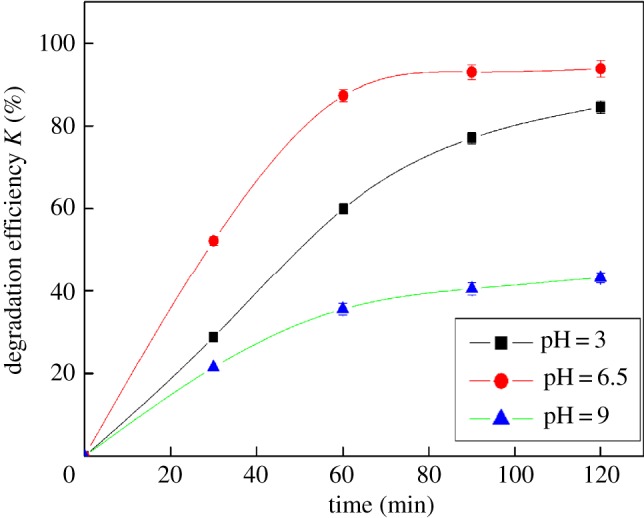
Effect of pH on the degradation of DBP (13 mg l^−1^ DBP, 100 ml; 30% H_2_O_2_, 50 µl; Fe_2_O_3_, 30 mg; temperature, 25°C).

For the multi-phase photocatalytic reaction of semiconductors, the pH value of the solution is an important factor affecting the kinetics of catalytic reaction. Figure 5 shows the effect of pH on the degradation of DBP. The photocatalytic degradation rate of DBP in Fe_2_O_3_/H_2_O_2_/UV system was the fastest under neutral condition. The main mechanism is described as follows: (1) different pH values can change the charge properties of the catalyst, especially the oxide semiconductor, so it can affect the adsorption behaviour of organic molecules on the catalyst surface; (2) H^+^ or OH^−^ in the solution can combine with the photo-generated charge to produce highly active species; and (3) change of pH may lead to change of some organic structure, thereby it can change the ease of catalytic oxidation.

The degradation efficiency of DBP was lower under acidic condition, and the photocatalytic degradation efficiency was the lowest under alkaline condition. This might be related to the stability of DBP at different pH conditions and the effect of the products obtained by photocatalytic degradation of DBP. Under acidic condition, DBP was more susceptible to oxidative degradation to produce organic acids such as benzoic acid, carboxylic acid and CO_2_, which reduces the reaction rate of the whole degradation process. Under alkaline condition, H_2_O_2_ was very unstable and easily decomposed into H_2_O and O_2_, so that the H_2_O_2_ content became low, which might affect the formation of •OH. So the photocatalytic degradation rate became slower. Based on the results shown in figure 5, the optimal pH was that of neutral condition.

### 3.6. Effect of initial dibutyl phthalate concentration on degradation of dibutyl phthalate

In general, the photocatalytic degradation of organics involves the processes of the charge transfer of organics and reactive groups (electron–hole pairs, •OH, etc.) that take place on the surface of the catalyst. Therefore, the initial concentration of organics has also an influence on the photocatalytic degradation efficiency.

**Figure 6.**
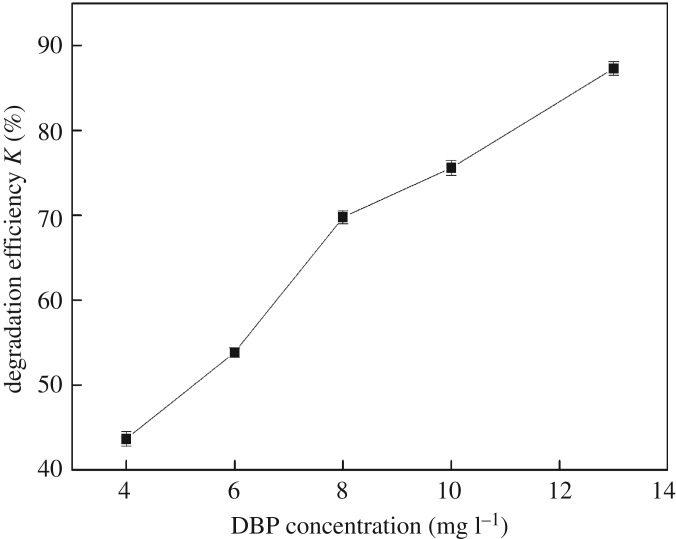
Effect of different initial concentration on the degradation efficiency (13 mg l^−1^ DBP, 100 ml; 30% H_2_O_2_, 50 µl; Fe_2_O_3_, 30 mg; temperature, 25°C).

Figure 6 shows the effect of different initial concentration on the degradation efficiency of DBP. The degradation rate increased with the initial concentration in the range of solubility of DBP. When the initial concentration was 13 mg l^−1^, the photocatalytic degradation efficiency was the highest. As the maximal solubility of DBP in water was 13 mg l^−1^, 13 mg l^−1^ of DBP solution was selected for photocatalytic degradation in this study.

### 3.7. Photocatalytic degradation process and mechanism of dibutyl phthalate

GC-MS not only can determine the molecular structure of a compound, and but also accurately determine the relative molecular weight of unknown components. In order to further study the photocatalytic degradation process and mechanism of DBP under high-pressure mercury lamp irradiation for 2 h and 12 h, the corresponding degradation intermediates of DBP were analysed by GC-MS.

Electronic supplementary material, figures S3–S13, show the GC-MS of DBP and main degradation intermediates. The corresponding retention time was 14.965, 14.805, 14.369, 13.133, 12.673, 12.211, 10.936, 10.316, 10.022 and 7.266 min, respectively. The analytic results are listed in table 1. Based on these results, the probable photocatalytic degradation pathway of DBP is shown in figure 7, and the probable reaction process and degradation mechanism were proposed as follows.

**Figure 7.**
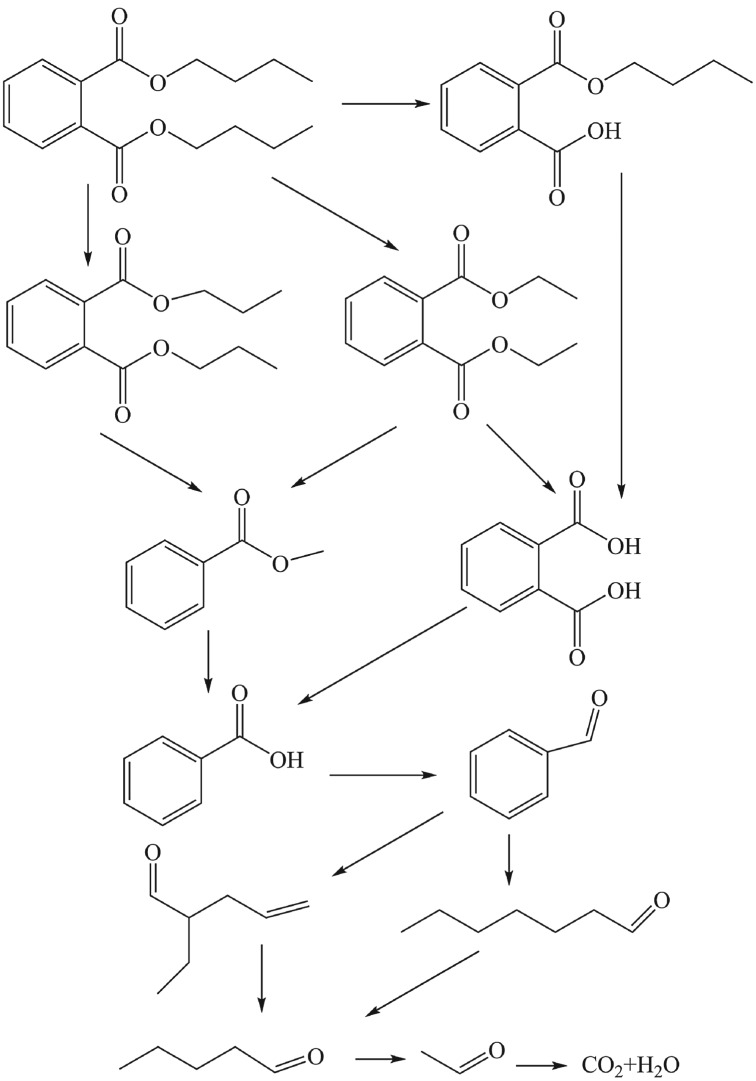
Photocatalytic degradation mechanism of DBP.

**Table 1.** The main intermediate photocatalytic degradation products of DBP.

retention time (min)	products	structure	molecular weight
14.965	dibutyl phthalate	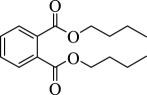	278
14.805	2-(butoxycarbonyl)benzoic acid	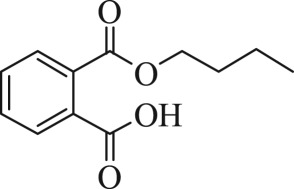	222
14.369	dipropyl phthalate	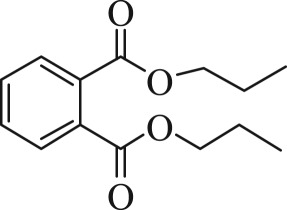	250
13.133	diethyl phthalate	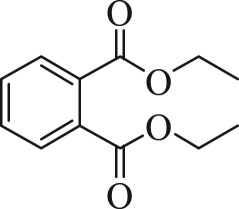	222
12.673	methyl benzoate	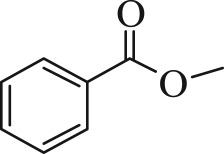	136
12.211	benzoic acid	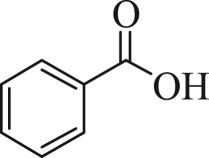	122
10.936	benzaldehyde	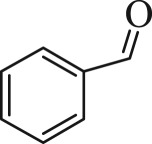	106
10.316	heptanal	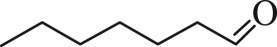	114
10.022	2-ethylpent-4-enal	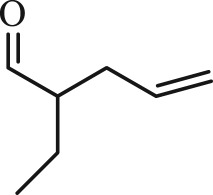	112

In the process of reaction, the electron–hole pairs generated from the excitation of photocatalyst α-Fe_2_O_3_ nanoparticles were transferred to the surface of the catalyst and combined with OH^−^, O_2_, H_2_O, etc. and adsorbed on the surface of the catalyst with the occurrence of energy and charge exchange, showing a strong oxidizing ability of hydroxyl radicals •OH, etc. First, the ester of the CO bond and the carbon chain of DBP in the solution were attacked by •OH to produce mono-butyl phthalate, phthalate and alkane moiety. Second, the carbon chain from different locations might be broken and the main products such as diethyl phthalate and dipropyl phthalate appeared. Under the action of •OH, phthalic acid and methyl benzoate formed benzoic acid. The C–O bonds in the carboxylic acid and carboxyl group were fractured and oxidized to generate benzaldehyde and then benzene formaldehyde was attacked by •OH, forming the heptaldehyde and 2-ethyl-4-pentenal. It could be seen that there were many possibilities for the ring-opening reaction of the benzene ring. The active group •OH attacked the terminal alkene group, resulting in valeraldehyde. Finally, CO_2_ and H_2_O could be produced by •OH.

## 4. Conclusion

In this study, α-Fe_2_O_3_/H_2_O_2_/UV was used as the photocatalytic degradation system of DBP. The optimum experimental conditions were obtained, and the maximum degradation efficiency could reach 94% when the hollow α-Fe_2_O_3_ dosage, 30% H_2_O_2_ content, pH value and initial DBP concentration were 300 mg l^−1^, 50 µl, 6.5 and 13 mg l^−1^, respectively. DBP could be degraded by the attack of active hydroxyl radicals, producing the corresponding intermediates, such as monobutyl phthalate, diethyl phthalate, methyl benzoate, benzoic acid, benzaldehyde, heptaldehyde, 2-ethyl-4-pentenal, during the reaction for 2 h. The DBP and its intermediates were almost completely degraded to mineralize to CO_2_ and H_2_O. These results illustrated that hollow α-Fe_2_O_3_ nanoparticles have great potential as catalyst to process more organic pollutants in the environment and provide technical support for the further study of the photocatalytic degradation of other organic pollutants.

## Supplementary Material

Supporting information

## Data accessibility

This article does not contain any additional data.
