# Supplementary material for: Photocatalytic degradation properties of α-Fe2O3 nanoparticles for dibutyl phthalate in aqueous solution system
Source: R Soc Open Sci. 2018 Apr 11;5(4):172196. doi: 10.1098/rsos.172196 (PMC5936939; doi:10.1098/rsos.172196)
Supplement: Supporting information [file rsos172196supp1.doc]

**Supporting information**

**
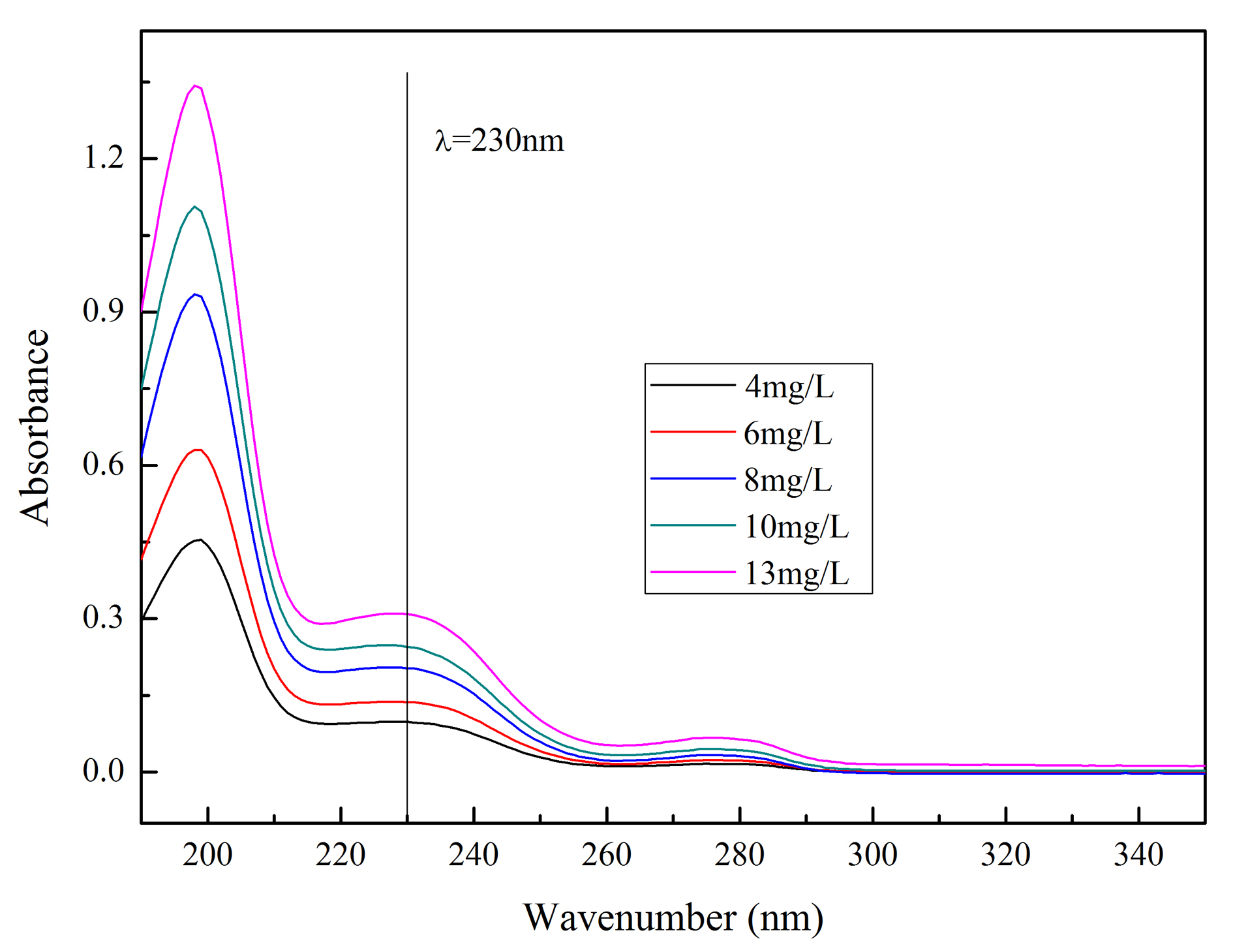
**

Fig.S1 The UV-Vis spectra of the different concentrations of DBP solution

**

**Fig.S2 The relationship between the mass concentration of DBP solution and the absorbance


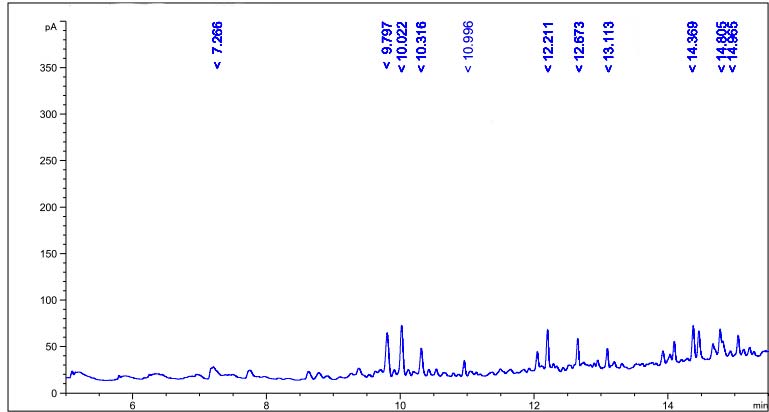


Fig.S3 Gas chromatograma of photodegradation mixutures of DBP for 2h


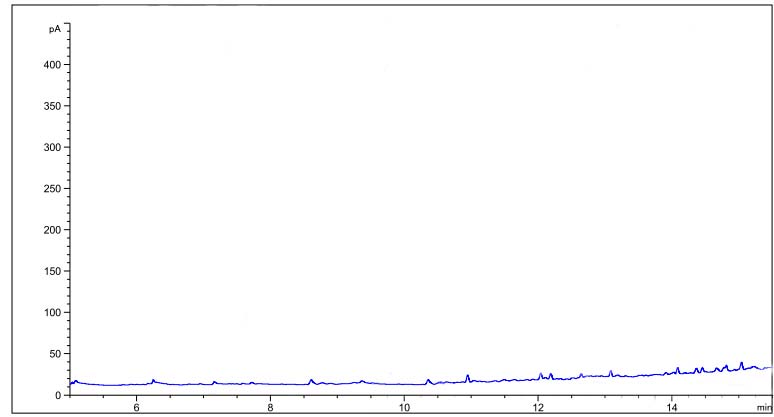


Fig.S4 Gas chromatograma of photodegradation mixutures of DBP for 12h


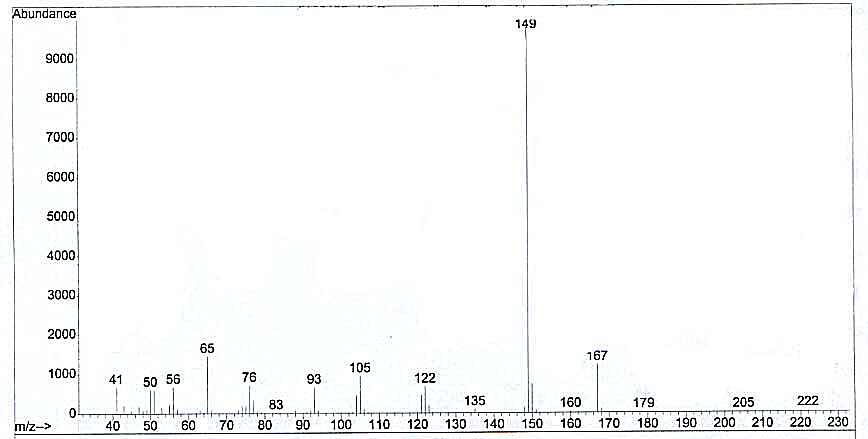


Fig. S5 Gas chromatogram of DBP(R=14.965)

**
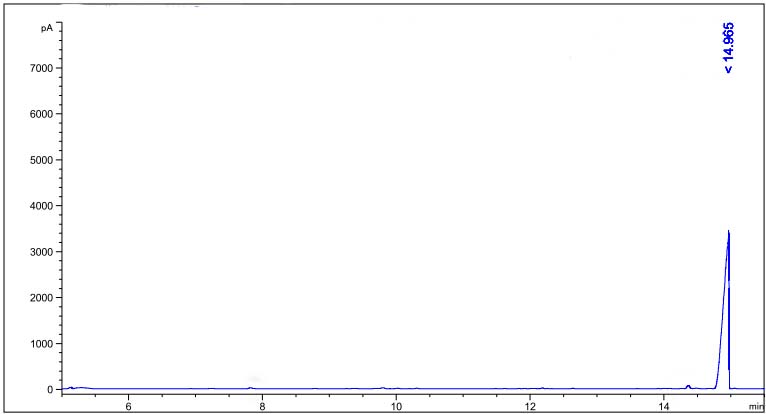
**

Fig. S6 The mass spectrum of the material with the retention time of 14.805 min


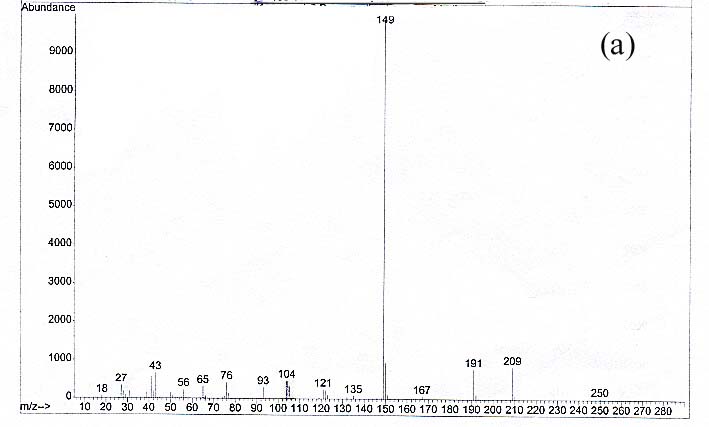


Fig. S7 The mass spectrum of the material with the retention time of 14.396 min


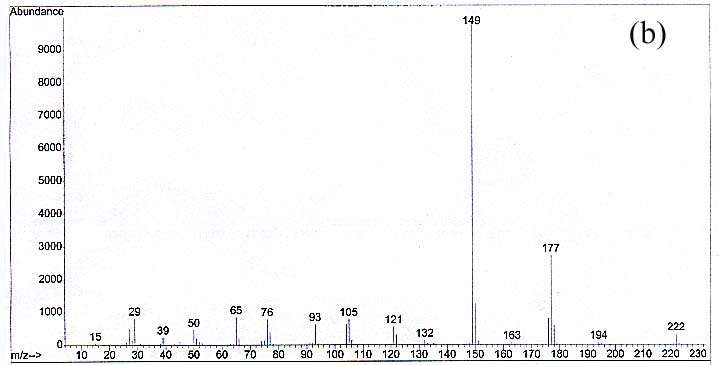


Fig. S8 The mass spectrum of the material with the retention time of 13.133 min


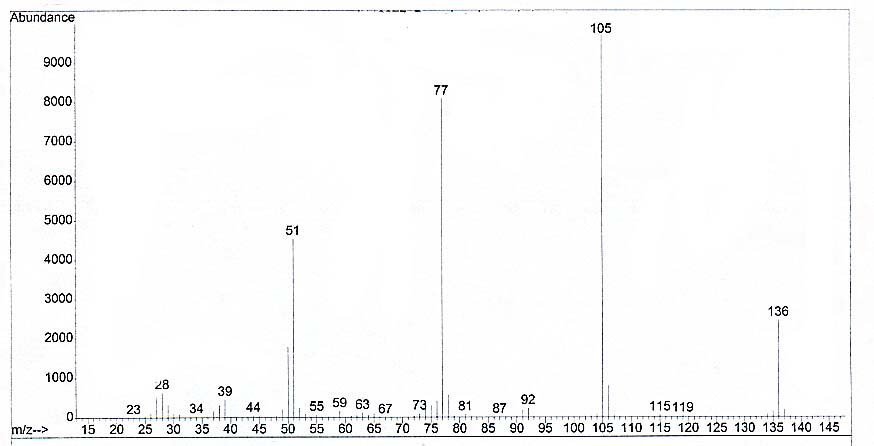


Fig. S9 The mass spectrum of the material with the retention time of 12.673 min


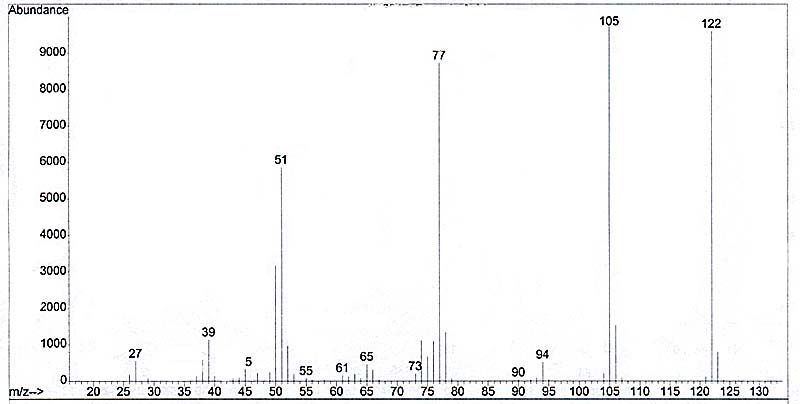


Fig. S10 The mass spectrum of the material with the retention time of 12.211 min


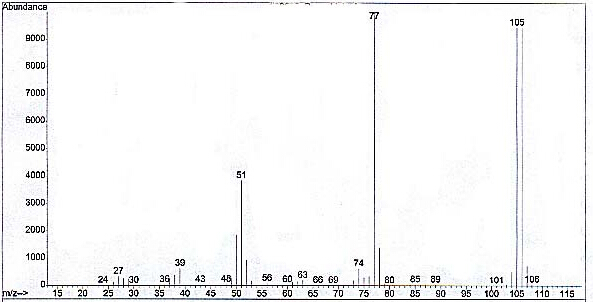


Fig. S11 The mass spectrum of the material with the retention time of 10.936 min


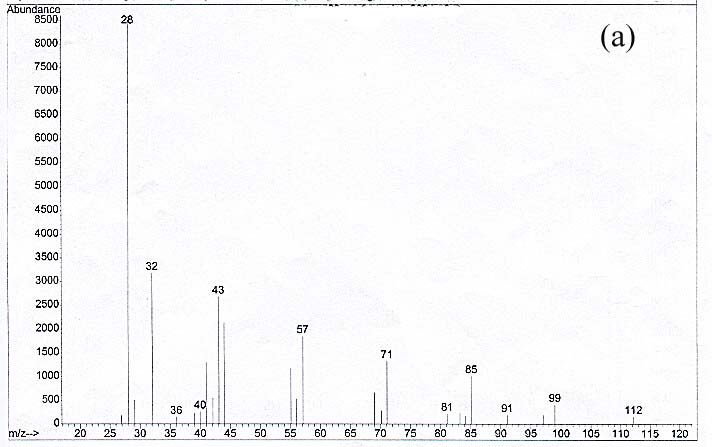


Fig. S12 The mass spectrum of the material with the retention time of 10.316 min


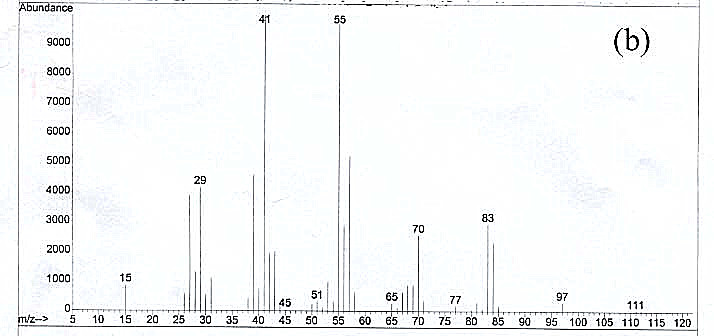


Fig. S13 The mass spectrum of the material with the retention time of 10.022 min
